# *omeSOM: a software for clustering and visualization of transcriptional and metabolite data mined from interspecific crosses of crop plants

**DOI:** 10.1186/1471-2105-11-438

**Published:** 2010-08-26

**Authors:** Diego H Milone, Georgina S Stegmayer, Laura Kamenetzky, Mariana López, Je Min Lee, James J Giovannoni, Fernando Carrari

**Affiliations:** 1 Research Center for Signals, Systems and Computational Intelligence, FICH-UNL, CONICET, Ciudad Universitaria UNL, Santa Fe, (3000), Argentina; 2 Centro de Investigación en Ingeniería en Sistemas de Información, CONICET, Lavaise 610, Santa Fe, (3000), Argentina; 3 Instituto de Biotecnología, Instituto Nacional de Tecnología Agrícola (IB-INTA), CONICET, PO Box 25, B1712WAA Castelar, Argentina (partner group of the Max Planck Institute for Molecular Plant Physiology, Potsdam-Golm, Germany; 4 Boyce Thompson Institute for Plant Research, Cornell University Campus, Ithaca, NY 14853, USA

## Abstract

**Background:**

Modern biology uses experimental systems that involve the exploration of phenotypic variation as a result of the recombination of several genomes. Such systems are useful to investigate the functional evolution of metabolic networks. One such approach is the analysis of transcript and metabolite profiles. These kinds of studies generate a large amount of data, which require dedicated computational tools for their analysis.

**Results:**

This paper presents a novel software named *omeSOM (transcript/metabol-ome Self Organizing Map) that implements a neural model for biological data clustering and visualization. It allows the discovery of relationships between changes in transcripts and metabolites of crop plants harboring introgressed exotic alleles and furthermore, its use can be extended to other type of omics data. The software is focused on the easy identification of groups including different molecular entities, independently of the number of clusters formed. The *omeSOM software provides easy-to-visualize interfaces for the identification of coordinated variations in the co-expressed genes and co-accumulated metabolites. Additionally, this information is linked to the most widely used gene annotation and metabolic pathway databases.

**Conclusions:**

*omeSOM is a software designed to give support to the data mining task of metabolic and transcriptional datasets derived from different databases. It provides a user-friendly interface and offers several visualization features, easy to understand by non-expert users. Therefore, *omeSOM provides support for data mining tasks and it is applicable to basic research as well as applied breeding programs. The software and a sample dataset are available free of charge at http://sourcesinc.sourceforge.net/omesom/.

## Background

At present, there is a data explosion in the biological sciences. A series of technical advances in recent years have led to an increase in the amount of data that biologists can recover concerning many aspects of an organism, both at genomic and post-genomic levels. Discovery of hidden patterns of gene expression in plants of economic importance to agro-biotechnology may aid in improving the quality of crop products. In addition, transcript and metabolite integration is gaining importance given the need for extracting knowledge from multiple data types and sources, with the aim of finding informative relations to infer new insights concerning the genetic processes underlying them [1-3]. In plant experimental biology and crop breeding, widely used systems include introgression lines and recombinant inbred lines (ILs and RILs, respectively), characterized genotypes carrying exotic alleles from related species. Although ILs and RILs have proven useful tools in crop domestication and breeding since time immemorial, their applicability as experimental systems exposing thousands of quantitative trait loci has become increasingly popular in recent years [4-6]. The effects on gene expression and metabolite accumulation in each line may provide important clues regarding the genes and metabolic pathways impacted by the introgressed segments [7]. A recent advance in this field has reported probabilistic associations and visualizations of genes, metabolites and phenotypes for such datasets [8]. Bioinformatics is playing an important role, allowing biologists to make full use of the advances in computer science for analyzing large and complex datasets.

Biological data sets in the omics era have several common problems: they are typically large, have inherent biological complexity, may have significant amounts of noise and may change with time requiring proper tracking. These challenges require novel design and adaptation of computer science techniques and models. Also, given the rapid expansion of biological data and the tools to handle them, there is both an increasing need and opportunity to extract information that may not have been obvious using past analysis methods. Large databases may contain interesting patterns that, if identified and validated by further laboratory work, can lead to novel discovery [9].

Bioinformatics has evolved mainly from the development of data mining techniques and their application to automatic prediction and discovery of classes [10]. The prediction of classes uses the information available from expression profiles and known features of the data and/or experiments to build classifiers for further data interpretation. Here we focus primarily on class discovery, where data are explored from the perspective that previously unknown relations can be identified and could lead to the formulation of novel hypotheses [11]. Two distinct types of class discovery methods exist: supervised, which are guided by a few hypotheses to be tested; and unsupervised, where no target variable is identified a priori and the mining algorithm searches for structures among all variables. The most common unsupervised data mining method is clustering [12]. Clustering refers to the grouping of observations or samples into classes of similar objects (named clusters) [13]. These algorithms segment the entire dataset trying to maximize the similarity of the samples within a cluster, minimizing their similarity to outside members [14]. For the analysis of these biological data, clustering is implemented under the assumption that behaviorally similar samples may be related to common pathways. According to this principle, named guilt-by-association, a set of genes involved in a biological process is co-expressed under the control of the same regulatory network [15]. It is presumed that if a gene with unknown function is co-expressed with a gene with known function participating in a recognised metabolic pathway it can be inferred that the unknown gene is also likely to be involved in the same pathway (for a review see [16]). Similar reasoning can be applied to analysis of metabolites and to the integration of both types of data.

Due to the limitations of traditional algorithms, computational intelligence has been recently applied to bioinformatics with promising results [17]. This research area includes artificial neural networks, evolutionary algorithms and fuzzy systems, each of them having its own characteristics and significant history. However, their application to bioinformatics problems remains a recent development [18]. In particular, artificial neural networks have been recently stressed as suitable for the task of clustering and knowledge discovery, for example the Self-Organizing Maps (SOMs) [19]. These neural models have proven to be adequate for handling large data volumes and projecting them in low dimensional maps while showing, at the same time, previously unknown relationships [20].

SOMs have been used for unsupervised clustering of transcriptome profiles increasingly over the past decade [21,22]. For example, GenePattern [23] provides support for several categories of gene expression analysis such as differential expression and selection, pathway analysis and class prediction/discovery through clustering. GenePattern supports SOMs as well as several traditional clustering methods, such as hierarchical clustering. Its use [24] in an earlier version called GeneCluster has indicated significantly regulated genes over time. More recently, AutoSOME [25] has been presented as a new method for automatically clustering SOM ensembles of high-dimensional data, such as that from whole genome microarrays.

Regarding metabolites, in [26] a correlation network analysis has revealed a sequential reorganization of metabolic and transcriptional states during germination and revealed gene-metabolite relationships in Arabidopsis. In [27] SOM clustering is used for the analysis of Arabidopsis thaliana metabolome and transcriptome datasets, helping in the hypothesis validation of a metabolic mechanism responding to sulfur deficiency. The results obtained after examination of each cluster by hand indicated that functionally related genes were clustered in the same or neighboring neurons.

In many cases, however, the biological experiment does not involve time or developmental change of a particular condition within a given genotype; rather genotypic differences form the basis of differential gene expression and metabolite accumulation. For example, it may involve an original genome that has been modified by introgression of wild species alleles (cisgenic plants) or transgenic plants expressing a gene of interest. Furthermore, the focus may be the identification of meaningful biological points (markers) that are hidden within large-scale analytical intensity measurements from metabolomic experiments. In [28] we have proposed a SOM model for finding relationships among ILs compared to a wild type control (IL-SOM) at a given developmental stage in contrast to genotype-specific data representing a time-course. Furthermore, the proposed model is oriented towards discovering new relationships among transcriptional and metabolic data, instead of verifying an a-priori condition.

For all of these tasks, many software tools implementing the use of SOMs have recently been presented. MarVis [29] performs data mining on intensity-based profiles using one-dimensional self-organizing maps. It has been developed for metabolome analysis, but it can also be applied to gene expression experiments. Simple BL-SOM [30] sets a SOM model for following the evolution of a previously-established condition over time. Vanted [31] is mainly presented as a tool for visualization of networks with related experimental data from large-scale biochemical experiments. Additionally, this tool uses a SOM for clustering the input data files according to similar behavior over time.

In this paper, we present the *omeSOM software, which trains a two-dimensional SOM for the analysis and interpretation of large amounts of data of different types such as gene expression and metabolite profiling. The analysis is performed over their genotypic differences instead of time evolution. The raw dataset used to test this software were derived from ripe tomato fruits harvested from a population of introgression lines derived from a cross between the tomato domesticated species *Solanum lycopersicum *and its wild relative *Solanum pennellii *[32]. The high variation in metabolite and transcript accumulations displayed by this kind of genetic material prompted us to select it to test the feasibility of using this software on these data. This work adds a new analytical dimension providing a specialized tool for data exploration, as well as for grouping and searching for new relationships between metabolites and transcripts. Furthermore, this software could be used to analyze many different types of omics data.

With *omeSOM we provide simple visualization interfaces for the identification of co-expressed and co-accumulated genes and metabolites at a glance, in a way that neurons grouping both types of data together are quickly highlighted. The focus of *omeSOM is on the easy identification of groups and pattern types, independently from the large collection of formed clusters.

The paper is structured as follows: first, implementation and software features are described followed by a discussion of the *omeSOM clustering. The visualization tools and a final discussion of biological applications round out the presentation.

## Implementation

The *omeSOM software has been implemented in the Matlab^® ^programming language. We used a standard toolbox for SOM training, provided by the original developers of this neural network model [33]. The software packages and documentation can be downloaded from the project home page http://sourcesinc.sourceforge.net/omesom/.

The *omeSOM software provides the following main options:

• Create *omeSOM model: creating an *omeSOM model requires an input file with the *.data *extension, for example *datasetname.data *(a detailed explanation of the required format file is given below). The map size should be typed by the user in the command line.

• **Search**: any input data point can be located on *omeSOM. This function returns the neuron number where a given metabolite name/transcript code has been grouped.

• **Neurons map**: several views of a trained map are possible, showing transcript (red), metabolite (blue) and both molecular entities (black) grouped into neurons. Detailed plots of normalized and un-normalized data are shown. Additionally, in the case of transcripts, their corresponding Arabidopsis [34] and Solanaceae Unigene [35] annotations can be retrieved. Also, a list of metabolic pathways [36] associated with each metabolite is shown.

• **3-colors map**: a specific view of the map is shown, painting the neurons according to a color scale that easily indicates those grouping transcripts and metabolites which are 1 standard deviation out of the neuron mean.

• **Neurons error measure**: a typical measure of clustering quality (cohesion) is calculated for each neuron and shown graphically over the feature map with different marker sizes.

• **Neurons having pseudo-zeros**: there are special situations where some metabolite may show undetectable levels in a specific genotype, having however valid measurements for many others.

The features described above constitute the fundamental functions of the software, which are constantly extended according to the users feedback.

## Results and Discussion

The case study used to test the *omeSOM software applicability involves the analysis of fruit transcriptome and metabolite profiles from a set of tomato ILs derived from a cross between *Solanum lycopersicum *and its wild relative *Solanum pennelli*. An example dataset can be downloaded from the project home page http://sourcesinc.sourceforge.net/omesom/.

### IL-dataset input file

Table 1 shows an example of an input dataset appropriate for *omeSOM. The input matrix must have the following format: a first row with the number of genotypes studied; a second one may have a comment line enclosing the name of each genotype. From the third row on, each line must have the measurements (*x*) for each IL of a single molecule (*m *for metabolite, *t *for transcript).

**Table 1:** Input training set containing measurements for *T *transcripts and *M *metabolites from *P *genotypes.

P IL_1_	IL_2_	...	IL*_i_*	...	IL*_P_*	
$x_{1}^{t_{1}}$	$x_{2}^{t_{1}}$	...	$x_{i}^{t_{1}}$	...	$x_{P}^{t_{1}}$	Transcript1
$–x_{1}^{t_{1}}$	$–x_{2}^{t_{1}}$	...	$–x_{i}^{t_{1}}$	...	$–x_{P}^{t_{1}}$	Transcript1(inv)
$x_{1}^{t_{2}}$	$x_{2}^{t_{2}}$	...	$x_{i}^{t_{2}}$	...	$x_{P}^{t_{2}}$	Transcript2
$–x_{1}^{t_{2}}$	$–x_{2}^{t_{2}}$	...	$–x_{i}^{t_{2}}$	...	$–x_{P}^{t_{2}}$	Transcript2(inv)
⋮	⋮	⋱	⋮	⋮	⋮	...
$x_{1}^{T}$	$x_{2}^{T}$	...	$x_{i}^{T}$	...	$x_{P}^{T}$	TranscriptT
$–x_{1}^{T}$	$–x_{2}^{T}$	...	$–x_{i}^{T}$	...	$–x_{P}^{T}$	TranscriptT(inv)
$x_{1}^{m_{1}}$	$x_{2}^{m_{1}}$	...	$x_{i}^{m_{1}}$	...	$x_{P}^{m_{1}}$	Metabolite1
$–x_{1}^{m_{1}}$	$–x_{2}^{m_{1}}$	...	$–x_{i}^{m_{1}}$	...	$–x_{P}^{m_{1}}$	Metabolite1(inv)
$x_{1}^{m_{2}}$	$x_{2}^{m_{2}}$	...	$x_{i}^{m_{2}}$	...	$x_{P}^{m_{2}}$	Metabolite2
$–x_{1}^{m_{2}}$	$–x_{2}^{m_{2}}$	...	$–x_{i}^{m_{2}}$	...	$–x_{P}^{m_{2}}$	Metabolite2(inv)
⋮	⋮	⋱	⋮	⋮	⋮	...
$x_{1}^{M}$	$x_{2}^{M}$	...	$x_{i}^{M}$	...	$x_{P}^{M}$	MetaboliteM
$–x_{1}^{M}$	$–x_{2}^{M}$	...	$–x_{i}^{M}$	...	$–x_{P}^{M}$	MetaboliteM(inv)

*omeSOM input file format.

Original and inverted versions of all data samples are included in the example.

Each measurement is an average log value $\left(logR_{i}^{*}\right)$, where * stands for the metabolite or transcript at the genotype *i*, calculated from the relative measurements of the compounds studied for valid experiments, where there are measurements for at least two technical replicates. The resulting log ratios are normalized. For each pattern, the sum of the square of log ratios is set equal to 1 according to

(1) $$x_{i}^{*}=\frac{logR_{i}^{*}}{\sum_{j=1}^{P}{\left(logR_{j}^{*}\right)}^{2}}$$

where *P *is the total number of genotypes studied.

Several data integrations are possible. For example, before integration of two datasets, the plus/minus sign of one dataset can be reversed to obtain negatively correlated items. All possible relations are *direct relations *between transcripts (*t*) and metabolites (*m*): ↑*t *↔↑*m *(inverted sign ↓*t *↔↓*m*), ↑*t *↔↑*t *(inverted sign ↓*t *↔↓*t*) and ↑*m *↔↑*m *(inverted sign ↓*m *↔↓*m*); and *cross relations*: ↑*t *↔↓*m *(inverted sign ↓*t *↔↑*m*), ↑*t *↔↓*t *(inverted sign ↓*t *↔↑*t*) and ↑*m *↔↓*m *(inverted sign ↓*m *↔↑*m*). Moreover, from an input dataset with only the original data, the software can generate the inverted patterns automatically. The main input file for the *omeSOM should be named in the following manner:

*• omesom *<*dataversion *>_- _<*date *>*T* <*time* >_-_*oToM.data*: input data where <dataversion> indicates the version of the data file format and *oToM *indicates original transcriptes and original metabolites.

Once the main. *data *file has been loaded, the following files are automatically searched for and loaded from the same directory:

*• omesom *<*dataversion *>_- _<*comp *>_- _<*date *>*T *<*time *>_-_*ilexp.data*: markers for expressed ILs, where < comp > indicates the component type: *trs *for transcripts and *mts *for metabolites;

*• omesom *<*dataversion *>_-_*mts*_- _<*date *>*T* <*time* >_-_*nonormsq.data*: un-normalized metabolites;

*• omesom *<*dataversion *>_-_*trs*_- _<*date*>*T* <*time* >_-_*nolowess.data*: un-normalized transcripts;

For the case study, the metabolic data were obtained analyzing polar extracts of tomato fruits, through Gas Chromatography coupled to Mass Spectrometry (GC-MS). The peak intensities were normalized to the internal standards added and to the mass of the tissue sample processed [37]. The metabolite profiling technique used allows the identification of approximately 80 primary metabolic compounds [2]. Metabolite accumulation measurements are obtained from 4 to 6 replicates of an experiment. Metabolites that do not appear in at least two independent replicates are not considered for further analysis. For each metabolite in each IL, the log ratio of the mean of the valid replicates is calculated. In the selection step only metabolites with log ratio greater than 0.1 are kept for data integration and cluster analysis.

Transcriptional levels were obtained from TOM2 chips (long oligo arrays representing approximately 12,000 tomato unigenes) ordered into spots, previously marked by hybridization with two fluorescence probes. Poor quality spots, negative spots, spots not expressed in both channels and empty spots were filtered out. Non-expressed spots were detected for each IL and control slide. Spots having a foreground signal mean less than the spot mean background plus two times the spot background standard deviation were selected. Spots with at least two replicated data points are included for analysis. These measures are then normalized using the print-tip Lowess normalization strategy [38] and the valid replicates were averaged. A total of *P *= 21 ILs were analyzed, with introgressions in chromosomes: 1, 2, 3, 5, 8, 10, 11 and 12. After pre-processing and selection steps, *M *= 71 metabolites and *T *= 1385 transcripts reached the threshold value to be considered valid data. For further details on the preprocessing and selection steps see [28].

### *omeSOM Clustering

Neural network-based clustering is closely related to the concept of competitive learning, which is based on the idea of units (neurons) that compete to respond to a given subset of inputs. The nodes in the input layer admit input patterns and are fully connected to the output nodes in the competitive layer. Each output node corresponds to a cluster and is associated with a prototype or weight vector. Given an input pattern, its distance to the weight vectors is computed and only the neuron closest to the input becomes activated. The weight vector of this winning neuron is further moved towards the input pattern. This competitive learning paradigm is also known as winner-takes-all learning [39]. Self-organizing maps (SOMs) represent a special class of neural networks that use competitive learning. Their aim is to represent complex high-dimensional input patterns in the form of a simple low-dimensional discrete map, with neurons that can be visualized in a two-dimensional lattice structure, while preserving the proximity relationships of the original data as much as possible [20]. Therefore, SOMs can be appropriate for cluster analysis when looking for underlying or so-called hidden patterns in data. A neighborhood function is defined for each neuron and when competition among the neurons is complete, SOMs update a set of weight vectors within the neighborhood of the winning neuron.

The *omeSOM software builds a SOM model oriented towards discovering unknown relationships among transcriptional and metabolite data, showing previously unknown clusters of coordinated up-regulated and down-regulated patterns in each tomato genotype. Several model topologies, map sizes and initialization strategies are possible. The initial vectors are set by principal component analysis, obtaining a learning process independent of the order of input of vectors, and hence reproducible. The model learning method is the batch training algorithm [20], where the whole training set is gone through at once and only after this the map is updated with the net effect of all the samples. Comparison between each pattern x* and each neuron weight vector **w***_j _*is measured through the standard Euclidean distance *d*(x*,**w***_j_*) = ||x* - **w***_j_*||_2_. We use a gaussian neighborhood function of the form $g_{ij}=e^{-\frac{\delta_{ij}^{2}}{2r^{2}}}$, where *δ_ij _*is the distance between neuron *i *and neuron *j *on the map grid and *r *is the neighborhood radius.

### *omeSOM Visualizations

An appropriate visualization of the resulting characteristics map, painting the neurons according to the type of data grouped, is proposed for helping in the rapid identification of combined data types. The setting of several possible visualization neighborhoods (*Vn*) of a neuron is also helpful for the easy detection of groups of combined data types, avoiding the need for an identification procedure. When a *Vn *is defined, all the neurons in the neighborhood of radius *Vn *are considered as a group and treated altogether accordingly.

For the special case of the *omeSOM, many interesting representations of clusters can be obtained from the projection of the patterns in the lattice of neurons. If the dataset includes the original data and all the data with inverted sign, the resulting map shows a symmetrical "triangular" configuration. This means that the top-right and down-left zones of the map group exactly the same data but have opposite sign. It can be seen directly from the data visualisation which genes and metabolites are up-regulated and down-regulated together or with the inverse relationship (down regulated genes grouped together with up-regulated metabolites). There is a specific zone in the map where the exactly opposite behavior for each IL can be found, which is useful for associating specific genes/metabolites to a specific genotype.

In a standard SOM, clusters are recognized as a group of nodes rather than considering each node as a cluster. The identification of clusters is mainly achieved through visualization methods such as the U-matrix [40]. This method computes the average distance between the codebook vectors of adjacent nodes, yielding a landscape surface where light colors stand for a short distance (a valley) and dark colors for longer distances (a hill). Then, the number of underlying clusters must be determined by visual inspection.

The visualizations provided by the proposed *omeSOM model, instead, provide a simple interface for helping in the rapid identification of co-expressed genes and co-accumulated metabolites via a simple color code. The focus is on the easy identification of groups of different patterns, independently of the number of neurons in a cluster. Furthermore, setting of several possible visualization neighborhoods (*Vn*) for a given neuron is also helpful for the easy detection of groups of combined data types, avoiding the need for the identification of neuron clusters. When a *Vn *is defined, all the neighboring neurons (according to the neighborhood radius set) are considered as a group and treated altogether accordingly, also for counting whether metabolites and transcripts are grouped.

The following visualizations are supported by *omeSOM:

#### Easy identification of clusters of combined data types

**Figure 1:**
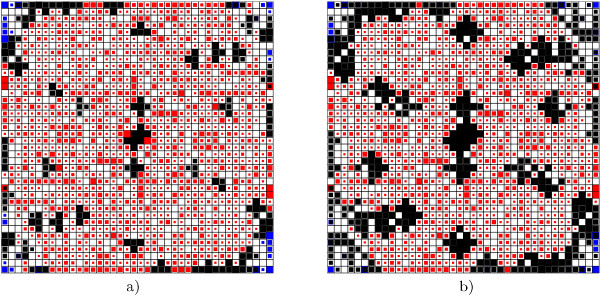
Main *omeSOM maps. Figure **1: **Activation *omeSOM resulting from the integrated analysis of 1385 genes and 71 metabolites from 21 tomato ILs. Map topology of 40 × 40 neurons with a) *V n *= 0 and b) *V n *= 1

Figure 1 shows different marker colors which indicate the kind of pattern grouped in the neuron: black for combined data types, blue for metabolites and red for transcripts that are grouped alone, with the option of setting these parameters in a black and white color scale. Also the marker size indicates the relative number of patterns grouped. In the neuron maps, when spots are selected they stay green after selecting another spot, to indicate to the user which neuron has already been analysed in detail. The figure shows the activation map resulting from the integrated analysis of 21 tomato ILs with a 40 × 40 neuron topology, with *Vn *= 0 and *Vn *= 1.

#### Detail view of original data measurements

**Figure 2:**
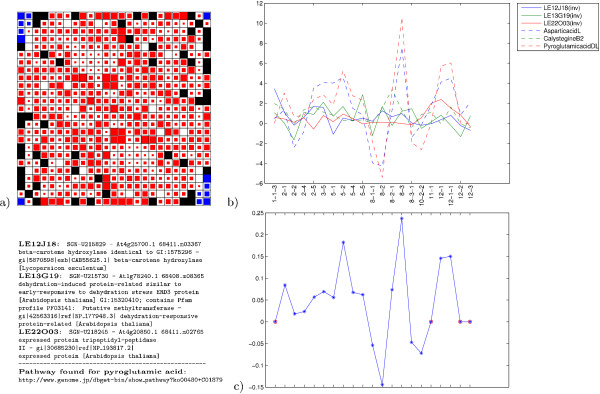
**Integration model visualizations**. Figure 2: (a) Resulting *omeSOM integrated model for the 21 ILs having 25 × 25 neurons with *V n *= 0. (b) Detail of the normalized cluster patterns values clustered together in neuron 604. (c) Detail of the de-normalized (original) values for the metabolite *pyroglutamic acid*. Down left panel: transcript codes decodification and metabolite pathways.

Figure 2 shows the resulting *omeSOM integrated model for the 21 IL dataset (a), with the same color code as that shown in Figure 1. Curves presented in b show a detail of the normalized patterns which have all been clustered together in neuron 604: three metabolites: *L-aspartic acid*, *calystegineB2 *and *D/L-pyroglutamic acid*, together with three inverted sign transcripts: *LE12J18*, *LE13G19 *and *LE22O03*. Figure 2 c shows the non-normalized (original) log ratios of *pyroglutamic acid*. Red circles indicate missing values for metabolites (samples in an IL not having enough significantly detected replicate experiments for the average log ratio calculation). In the case of a transcript, red circles indicate non-significant expression levels with respect to the control genotype. The lower-left panel shows the annotation of one transcript according to its probe code which is automatically linked into the Arabidopsis (At) and Unigene (SGN-U) annotations and metabolite pathways. A screenshot of these figures in the software can be found in the Additional file 1: Supplemental Figure S1.

#### KEGG pathways associated with grouped compounds

If metabolites and transcripts are named consistently with the Kyoto Encyclopedia of Genes and Genomes (KEGG) [41] conventions, data grouped by neurons can be checked against metabolic pathways available online, for finding candidate genes belonging to metabolic pathways. For each metabolite, a list of KEGG pathways where it participates can be easily visualized in the same interface. The software performs cross-references searches inside KEGG to obtain the corresponding pathway descriptions, using the metabolite KEGG codes.

#### Visualization of clusters inside a specific chromosome segment

**Figure 3:**
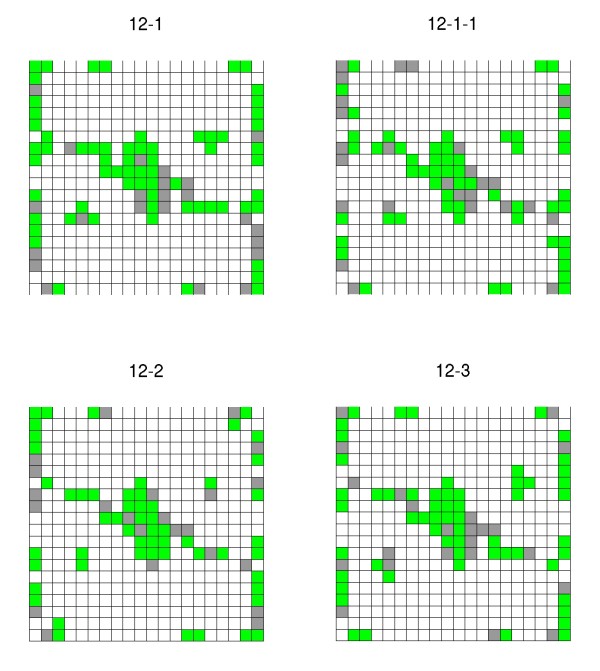
**3-colors maps example**. Figure 3: 3-colors maps activation for the tomato chromosome 12 (ILs 12-1-1, 12-1, 12-2 and 12-3). In gray: mean transcripts and/or metabolites which are below 1 standard deviation out of the neuron mean. In green: mean transcripts and/or metabolites which are above 1 standard deviation out of the neuron mean. In other cases, the neurons are painted white.

Another possibility is the visualization of clusters from all ILs belonging to the same chromosome. It is possible to select the data from a particular chromosome from a full genome dataset. This allows users to dissect particular clustering patterns showed by ILs spanning a given chromosome. When components of a neuron (transcript, metabolite or both) show significant deviations of the neuron mean with respect to the other ILs, they can be visualized in separated maps as those showed in Figure 3 for ILs spanning chromosome 12. This allows the comparison of pattern expressions according to a color scale that paints only neurons having patterns with an important deviation from the neuron mean, for each dimension/IL. That is, neurons where at least one pattern has a value greater than the mean plus one standard deviation in the corresponding IL are depicted in green. If in this IL there is at least one pattern in the neuron with a value lower than the mean plus one standard deviation, the neuron is painted in gray. The variations in the gene expression levels and/or metabolite variation of the grouped patterns may provide useful information regarding genes/metabolites specifically associated with a certain introgressed chromosomal segment. This feature could help in the association of metabolite and transcript networks with genetic maps. Figure 3 presents the output of the 3-color map function which shows the activation of a 20 × 20 map for all ILs comprising chromosome 12. The comparison of these maps allows identifying those ILs showing distinctive neurons. This feature might facilitate mapping those genetic factors involved in the clusters.

#### Quality evaluation of clusters of combined data types

It is quite important to be able to evaluate the quality of a clustering algorithm when applied to biological data, in particular if later biological inferences should be made. Inside *omeSOM, a typical clustering measure is calculated for each neuron and shown graphically over the feature map with different marker sizes, when the feature Neurons error measure is selected. This measure comprises validation measures assessing cluster compactness or homogeneity [42]. Intracluster variance is their most popular representative:

(2) $${\bar{C}}_{j}=\frac{1}{\left|\Omega_{j}\right|}\sum_{\forall x_{i}\in \Omega_{j}}{\left\|x_{i}-w_{j}\right\|}_{2},$$

where |Ω*_j_*| is the number of patterns in node *j*. As a global measure of compactness, the average over all nodes is calculated $\bar{C}=\frac{1}{k}\sum_{j}{\bar{C}}_{j}$. Values of $\bar{C}$ close to 0 indicate more compact nodes.

## Conclusions

The *omeSOM model is oriented towards discovering unknown relationships among data, as well as providing simple visualizations for the identification of co-expressed genes and co-accumulated metabolites. It has a user-friendly interface and provides several visualization tools easy to understand by non-expert users. A case study which involved gene expression measurements and metabolite profiles from tomato fruits was presented here to show the application of the software. The interest in comparing the cultivated tomato against the different ILs lies on the fact that, as has been shown, some wild tomato relatives can be sources of important agronomical characters which could be used for the improvement of commercial tomato lines. Therefore, *omeSOM is presented as a software designed to give support to the data mining task applied to both basic research and applied breeding programs.

## Availability and requirements

• Project name: *omeSOM.

• Project home page: http://sourcesinc.sourceforge.net/omesom/.

• Operating system(s): Linux and Microsoft Windows.

• Programming language: Matlab^®^(release 2007a).

• Other requirements: SOM toolbox.

• License: opensource, free for academic use.

## Authors' contributions

DM and GS implemented the *omeSOM graphical user interface, the clustering algorithm and the clustering measurement, and wrote the manuscript. JG and JL provided the transcript case study dataset. LK, ML and FC provided the metabolite case study dataset, tested the software and wrote the manuscript. All authors read and approved the final manuscript.

## Supplementary Material

Additional file 1 *omeSOM screenshot. Several windows of the software are shown in the picture. The main menu (down right) shows the features provided by the software. By clicking on *Neurons map*, the upper left window appears showing a 2-D SOM model, where each neuron is painted according to the type of data it contains. Right-clicking on one neuron, let us suppose neuron 16, the upper right window appears showing a detail of the normalized patterns values that have been clustered together in neuron 16. Here, if one of the patterns is selected, the down right window appears, with detail of the de-normalized (original) values for the pattern; in this case, transcript *LE7E01*. In the background (down left) the corresponding transcript code decodification appears (Arabidopsis (At) and Unigene (SGN-U) annotations), as well as a list of related KEGG pathways. Click here for file
