# Supplementary figures and images for: *omeSOM: a software for clustering and visualization of transcriptional and metabolite data mined from interspecific crosses of crop plants
Source: BMC Bioinformatics. 2010 Aug 26;11:438. doi: 10.1186/1471-2105-11-438 (PMC2942854; doi:10.1186/1471-2105-11-438)

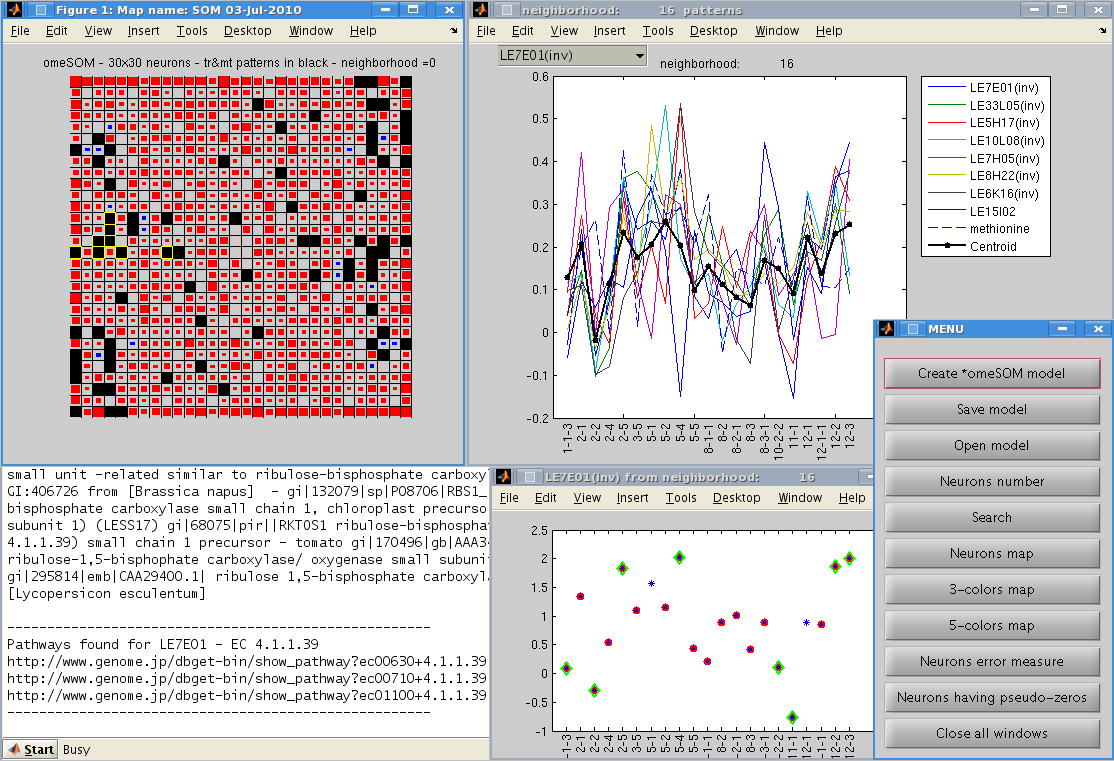

Supplement: Additional file 1 — *omeSOM screenshot. Several windows of the software are shown in the picture. The main menu (down right) shows the features provided by the software. By clicking on Neurons map, the upper left window appears showing a 2-D SOM model, where each neuron is painted according to the type of data it contains. Right-clicking on one neuron, let us suppose neuron 16, the upper right window appears showing a detail of the normalized patterns values that have been clustered together in neuron 16. Here, if one of the patterns is selected, the down right window appears, with detail of the de-normalized (original) values for the pattern; in this case, transcript LE7E01. In the background (down left) the corresponding transcript code decodification appears (Arabidopsis (At) and Unigene (SGN-U) annotations), as well as a list of related KEGG pathways. [file 1471-2105-11-438-S1.PNG]
